# Studies of Cream Seeded Carioca Beans (*Phaseolus vulgaris* L.) from a Rwandan Efficacy Trial: *In Vitro* and *In Vivo* Screening Tools Reflect Human Studies and Predict Beneficial Results from Iron Biofortified Beans

**DOI:** 10.1371/journal.pone.0138479

**Published:** 2015-09-18

**Authors:** Elad Tako, Spenser Reed, Amrutha Anandaraman, Steve E. Beebe, Jonathan J. Hart, Raymond P. Glahn

**Affiliations:** 1 USDA-ARS Robert W. Holley Center for Agriculture & Health, Cornell University, Ithaca, NY, 14853, United States of America; 2 Department of Food Science, Cornell University, Ithaca, NY, 14853, United States of America; 3 CIAT- International Center for Tropical Agriculture, Cali, 6713, Colombia; The Pennsylvania State University Hershey Medical Center, UNITED STATES

## Abstract

Iron (Fe) deficiency is a highly prevalent micronutrient insufficiency predominantly caused by a lack of bioavailable Fe from the diet. The consumption of beans as a major food crop in some populations suffering from Fe deficiency is relatively high. Therefore, our objective was to determine whether a biofortified variety of cream seeded carioca bean (*Phaseolus vulgaris* L.) could provide more bioavailable-Fe than a standard variety using *in-vivo* (broiler chicken, *Gallus gallus*) and *in-vitro* (*Caco-2* cell) models. Studies were conducted under conditions designed to mimic the actual human feeding protocol. Two carioca-beans, a standard (G4825; 58μg Fe/g) and a biofortified (SMC; 106μg Fe/g), were utilized. Diets were formulated to meet the nutrient requirements of *Gallus gallus* except for Fe (33.7 and 48.7μg Fe/g, standard and biofortified diets, respectively). *In-vitro* observations indicated that more bioavailable-Fe was present in the biofortified beans and diet (P<0.05). *In-vivo*, improvements in Fe-status were observed in the biofortified bean treatment, as indicated by the increased total-body-Hemoglobin-Fe, and hepatic Fe-concentration (P<0.05). Also, DMT-1 mRNA-expression was increased in the standard bean treatment (P<0.05), indicating an upregulation of absorption to compensate for less bioavailable-Fe. These results demonstrate that the biofortified beans provided more bioavailable Fe; however, the *in vitro* results revealed that ferritin formation values were relatively low. Such observations are indicative of the presence of high levels of polyphenols and phytate that inhibit Fe absorption. Indeed, we identified higher levels of phytate and quercetin 3–glucoside in the Fe biofortified bean variety. Our results indicate that the biofortified bean line was able to moderately improve Fe-status, and that concurrent increase in the concentration of phytate and polyphenols in beans may limit the benefit of increased Fe-concentration. Therefore, specific targeting of such compounds during the breeding process may yield improved dietary Fe-bioavailability. Our findings are in agreement with the human efficacy trial that demonstrated that the biofortified carioca beans improved the Fe-status of Rwandan women. We suggest the utilization of these *in vitro* and *in vivo* screening tools to guide studies aimed to develop and evaluate biofortified staple food crops. This approach has the potential to more effectively utilize research funds and provides a means to monitor the nutritional quality of the Fe-biofortified crops once released to farmers.

## Introduction

Iron (Fe) deficiency is the most common and widespread nutritional disorder in the world [1]. Fe deficiency is highly prevalent in low—income countries due to a lack of meat consumption in addition to a notable dietary reliance on grains containing high amounts of Fe absorption inhibitors (e.g., phytic acid, polyphenolic compounds) [2–4]. Major pathophysiological complications related to insufficient Fe intake may include stunted growth, impaired physical and cognitive development, and increased risk of morbidity and mortality in children [5–8]. To alleviate Fe deficiency, an integral step involves the understanding of specific dietary patterns and components that contribute to Fe status in the particular population suffering from a deficiency.

The common bean is a nutritious legume crop that is widely consumed by at-risk populations in Central and East Africa and Latin America [9, 10, 11]. In Rwanda, more than 50% of pre-school children are anemic [5, 7, 8]. Central to this crisis are low Fe intake, poor Fe absorption, and/or increased dietary Fe requirements to meet physiological needs (e.g., pregnancy) [8]. Biofortified staple food crops have become an effective tool by which to address micronutrient deficiencies, especially that of Fe, in many at-risk populations [4, 10]. Therefore, bean has been a target crop for Fe biofortification in Rwanda, as in many parts of Rwanda and western Kenya, the per capita bean consumption can reach as high as 66 kg/ year [10, 12–15].

Previous studies using Fe biofortified beans in Mexico have shown some improvement in Fe status in subjects consuming the biofortified beans versus a standard bean variety [11]. In addition, a recent clinical study conducted on Rwandese women using carioca beans [16], fractional Fe absorption from meals comprising the biofortified beans, with a Fe concentration of 8.8mg/100g, was 30% lower than from meals containing the standard beans with an Fe concentration of 5.4mg/100g. Although there was an increase in the overall Fe absorption by 19% from the biofortified bean meals, this increase was lower than expected since the Fe biofortified bean meals contained about 50% more Fe. Still, the Fe biofortified beans improved the Fe status in Rwandan University women, as after 135 days of feeding, there was a significant group difference in change in Hb and body Fe in the biofortified Fe bean group compared to control (standard) bean group, hence it was concluded that consumption of Fe-biofortified beans over 4.5 months improved Fe status in Fe depleted Rwandan women [17]. However, in addition to the increased Fe concentration provided by biofortified bean varieties, they have also been shown to contain higher amounts of phytic acid [16, 18], a well-studied inhibitor of Fe uptake *in vivo* [19–22]. It was also shown that a significant increase in Fe concentration in a biofortified bean variety does not always translate to a proportional increase in Fe bioavailability, as in addition to phytic acid polyphenolic compounds can be present in elevated quantities in biofortified staple food crops, such as beans, and these compounds may significantly reduce dietary Fe absorption [2, 18, 23–26]. Overall, there is evidence to suggest that both phytic acid and certain polyphenolics can significantly limit the nutritional benefit of increasing absorbable Fe in various staple food crops [2, 10, 16, 18, 23–29]. Hence, as was previously suggested, it is necessary to measure the concentration of Fe, the amount of bioavailable Fe, and the concentration of potential inhibitors of Fe bioavailability in these biofortified crops [2, 18].

The carioca beans used in the current study were developed at the International Center for Tropical Agriculture (CIAT) in Cali, Colombia. The biofortified beans are a mixture of high Fe sister lines that carry the SMC code (106μg Fe/g), whereas the standard-Fe bean, G4825 (57 μg Fe/g), is a commercial bean variety that was obtained from the Genetic Resources Unit of CIAT. Biofortified crops have become an effective tool by which to address micronutrient deficiencies, especially that of Fe, in many at-risk populations [4, 10, 13]. By using an *in vivo* (*Gallus gallus*) model that has been used extensively for nutritional research and shown to be an excellent animal to model Fe bioavailability [2, 18, 23, 30, 31], the objective of the current study was to compare Fe bioavailability in vitro and the ability of standard and Fe biofortified carioca bean lines to deliver Fe for Hb synthesis. If this *in vivo* assessment indicates that nutritional benefit exists (as was suggested by the Rwandan human efficacy bean trial) [16], we suggest to further employ these screening tools to guide future studies aimed to assess biofortified staple food crops, as this approach will allow proceeding to human efficacy studies with greater confidence and success.

## Materials and Methods

### Ethics statement

All animal protocols were approved by the Cornell University Institutional Animal Care and Use Committee (protocol name: *Intestinal uptake of Fe and Zn in the duodenum of broiler chicken*: *extent*, *frequency and nutritional implications*; protocol number: 2007–0129).

### Animals, Diets and Study Design

Cornish cross—fertile broiler eggs (n = 72) were obtained from a commercial hatchery (Moyer’s chicks, Quakertown, PA). The eggs were incubated under optimal conditions at the Cornell University Animal Science poultry farm incubator. Upon hatching (hatchability rate = 92%), chicks were allocated into 2 treatment groups on the basis of body weight, gender, and blood hemoglobin concentration (aimed to ensure equal concentration between groups, n = 14), 1) Fe Biofortified: 34.6% carioca bean based diet (48.7 ± 1.50 μg Fe/g), and 2) Standard Fe: 34.6% carioca bean based diet (33.7 ± 0.80 μg Fe/g). The two carioca bean lines used in this study were obtained from CIAT, and were shipped to Ithaca, New York in sealed containers imported as grain. Upon arrival, all beans were rinsed in ultra pure (18Ω) water and then cooked using an autoclave for 45 minutes in water and until soft. Beans were then freeze-dried and milled prior to mixing the diets (for all processing, stainless steel appliances were used). Experimental diets (Table 1) had no supplemental Fe. The specific Rwandese dietary formulation that was used in the study (Table 1) was achieved by a close consultation with and approval of the HarvestPlus nutritionist team, and was based on the menus that were used during the human efficacy trial [17]. Chicks were housed in a total confinement building (4 chicks per 1 m^2^ metal cage). The birds were under indoor controlled temperatures and were provided 16 h of light. Each cage was equipped with an automatic nipple drinker and a manual self—feeder. All birds were given *ad libitum* access to water (Fe content was 0.379 ± 0.012 ppm). Feed intakes were measured daily (as from day 1), and Fe intakes were calculated from feed intakes and Fe concentration in the diets.

**Table 1:** Composition of the experimental bean based diets^1^ ^–^ ^3^.

Ingredient	Fe content	Standard Bean Diet	Biofortified Bean Diet
	μg Fe/g, (n = 5, by analysis)	g/kg (by formulation)	
High-Fe Beans	106.1±0.204	–	346
Low-Fe Beans	57.10±0.145	346	–
Basmati Rice	0.290±0.006	135	135
Pasta (non-enriched)	11.48±0.358	70	70
Potato flakes	10.26±0.061	215	215
Banana Chips	7.510±0.521	115	115
Cabbage	16.32±0.400	30	30
Tomato powder	39.92±1.187	16	16
Orange sweet potatoes	26.90±0.611	73	73
Vitamin/mineral premix (no Fe)	0.00±0.00	70	70
DL-Methionine	0.00±0.00	2.5	2.5
Vegetable oil	0.00±0.00	30	30
Choline chloride	0.00±0.00	0.75	0.75
Total (g)		1000	1000
**Selected components**		**n = 5 (by analysis)**	
Dietary Fe concentration (μg/g)	–	33.7±0.80^b^	48.7±1.50^a^
Phytic acid (μg/g)	–	10605±742^b^	13793±1172^a^
Phytate:Fe molar ratio	–	15.43±0.85^a^	10.95±0.65^b^

^1^Vitamin and mineral premix provided/kg diet (330002 Chick vitamin mixture; 235001 Salt mix for chick diet; Dyets Inc. Bethlehem, PA).

^2^Iron concentrations in the diets were determined by an inductively-coupled argon-plasma/atomic emission spectrophotometer (ICAP 61E Thermal Jarrell Ash Trace Analyzer, Jarrell Ash Co. Franklin, MA) following wet ashing.

^3^Method for determining phytate is described in the materials and methods section.

^a,b^ Within a row, means without a common letter are significantly different (p < 0.05).

### Blood analysis, hemoglobin (Hb) determination, and tissue collection

Blood samples were collected weekly from the wing vein (n = 14, ∼100μL) using micro-hematocrit heparinized capillary tubes (Fisher, Pittsburgh, PA). Samples were collected in the morning following an 8 h overnight fast. Weekly blood Hb concentrations were determined spectrophotometrically using the cyanmethemoglobin method (H7506-STD, Pointe Scientific Inc. Canton, MI) following the kit manufacturer’s instructions.

Fe bioavailability was calculated as hemoglobin maintenance efficiency (HME) [2, 18, 23, 24, 30, 31]:

$$HME\;=\frac{Hb\;Fe,\;mg\;\left(final\right)-\;Hb\;Fe,\;mg\;\left(initial\right)}{Total\;Fe\;Intake,\;\;mg}\times 100$$

Where Hb—Fe (index of Fe absorption) = total body hemoglobin Fe. Hb—Fe was calculated from hemoglobin concentrations and estimates of blood volume based on body weight (a blood volume of 85 mL per kg body weight is assumed) [2, 18, 23, 24, 30, 31]:

Hb–Fe (mg) = B.W.(kg)×0.085 blood/kg ×Hb (g/L) ×3.35 mg Fe/g Hb

At the end of the experiment (day 42), birds were euthanized by CO_2_ exposure. The digestive tracts and livers were quickly removed from the carcass and separated into various sections for tissue analysis (small intestine and liver, ~ 1–2 cm and ~ 2–3 g, respectively). The samples were immediately frozen in liquid nitrogen, and then stored in a −80°C freezer until further analysis.

### Isolation of total RNA

Total RNA was extracted from 30 mg of duodenal (proximal duodenum) tissue using the Qiagen RNeasy Mini Kit (Qiagen Inc.,Valencia, CA) according to the manufacturer’s protocol. All steps were carried out under RNase free conditions. RNA was quantified by absorbency at 260–280 nm. Integrity of the 28S and the 18S rRNA was verified by 1.5% agarose gel electrophoresis followed by ethidium bromide staining [2, 18, 23, 24, 30–32].

### DMT–1, DcytB and, ferroportin gene expression analysis

As previously described [2, 18, 23, 24, 30, 31], PCR was carried out with primers chosen from the fragments of chicken duodenal tissues [DMT–1 gene (GeneBank database; GI 206597489) (forward: 5’-AGC CGT TCA CCA CTT ATT TCG-3’; reverse: 5’-GGT CCA AAT AGG CGA TGC TC-3’), DcytB gene (GI 20380692) (forward: 5’-GGC CGT GTT TGA GAA CCA CAA TGT T-3’; reverse: 5’-CGT TTG CAA TCA CGT TTC CAA AGA T-3’) and Ferroportin gene (GI 61098365) (forward: 5’-GAT GCA TTC TGA ACA ACC AAG GA’; reverse: 5’-GGA GAC TGG GTG GAC AAG AAC TC-3’). Ribosomal 18S was used to normalize the results (GI 7262899) (forward: 5’- CGA TGC TCT TAA CTG AGT-3’; reverse: 5’-CAG CTT TGC AAC CAT ACT C-3’)]. All PCR products were separated by electrophoresis on 2% agarose gel stained with ethidium bromide, and quantified using the Quantity One 1-D analysis software (Bio-Rad, Hercules, CA).

### *In*–*vitro* Fe bioavailability assessment

An *in vitro* digestion /Caco-2 cell culture model was used to assess in vitro Fe bioavailability [2, 18, 23, 24, 30, 31]. With this method, the cooked bean samples, additional meal plan components and the formulated diets were subjected to simulated gastric and intestinal digestion. 0.5 g of the freeze dried cooked beans and diet samples were utilized for each replication (n = 6) of the *in vitro* digestion process.

### Harvesting of Caco-2 cells for ferritin analysis

The protocols used in the ferritin and the total protein contents analyses of Caco-2 cells were similar to those previously described [2, 18, 23, 24, 30, 31]. Caco-2 cells synthesize ferritin in response to increases in intracellular Fe concentration. Therefore, we used the ratio of ferritin/total protein (expressed as ng ferritin/ mg protein) as an index of the cellular Fe uptake. All glassware used in the sample preparation and analyses was acid washed.

### Ferritin and Fe in the liver, electrophoresis, staining and measurement of gels

Liver ferritin and liver Fe quantification were conducted as previously described [2, 18, 23, 24, 30, 31]. The gels were scanned with a Bio-Rad densitometer, and measurements of the bands were conducted using the Quantity-One 1-D analysis program (Bio-Rad, Hercules, CA). All assays were conducted in duplicates for each animal in both biofortified and standard treatment groups (n = 14).

### Polyphenol extraction

Isolated beans seed coats were prepared by wrapping whole beans in de-ionized water-soaked paper towels until seed coats began to wrinkle and separate from cotyledons. Seed coats were then removed with forceps, dried, and ground to a coarse powder with mortar and pestle. To one gram of ground material, 8 mL of methanol:water (50:50 v:v) was added. The slurry was vortexed for one minute, placed on an orbital shaker for 25 minutes, then placed in a 30C sonication water bath for 15 minutes, vortexed again for one minute, and centrifuged at 4000 x g for 12 minutes. The supernatant was filtered with a 0.2 μm Teflon syringe filter and stored for later use in a -20C freezer.

### Ultra performance liquid chromatography—mass spectrometry (UPLC—MS) analysis of polyphenols

Seed coat extracts and polyphenol standards were analyzed with an Agilent 1220 Infinity UPLC coupled to an Advion expressionL compact mass spectrometer (CMS). 2 μL samples were injected and passed through an Acquity UPLC BEH Shield RP18 1.7 μm 2.1 x 100 mm column (Waters) at 0.35 mL/min. The column was temperature-controlled at 45C. The mobile phase consisted of water with 0.1% formic acid (solvent A) and acetonitrile with 0.1% formic acid (solvent B). Polyphenols were eluted using linear gradients of 86.7 to 77.0% A in 0.5 min, 77.0 to 46.0% A in 5.5 min, 46.0 to 0% A in 0.5 min, hold at 0% A for 3.5 min, 0 to 86.7% A in 0.5 min, and hold at 86.7% A for 3.5 min for a total 14 min run time. From the column, flow was directed into a variable wavelength UV detector set at 278 nm. Flow was then directed into the source of an Advion expressionL CMS (Advion Inc., Ithaca, NY) and ESI mass spectrometry was performed in negative ionization mode using selected ion monitoring with a scan time of 50 msec for each of 8 polyphenol masses of interest. Capillary temperature and voltages were 300C and 100V, respectively. ESI source voltage and gas temperature were 2.6kV and 240C respectively. Desolvation gas flow was 240 L/hr. LC and CMS instrumentation and data acquisition were controlled by Advion Mass Express software. Identities of polyphenols in bean samples were confirmed by comparison of m/z and LC retention times with authentic standards. Polyphenol quantification was achieved by the use of standard curves and integration of UV absorption peak areas.

### Determination of phytic acid concentration in the diet samples

Dietary phytic acid (phytate)/total phosphorus was measured as phosphorus released by phytase and alkaline phosphatase, following the kit manufacturer’s instructions (n = 5, K-PHYT 12/12, Megazyme International, Ireland).

### Statistical analysis

Results were analyzed by ANOVA using the general linear models procedure of SAS software (SAS Institute Inc. Cary, NC). Differences between treatments were compared by using a paired, two-tailed Student’s t—test and values were considered statistically different at p < 0.05 (values in the text are means ± SEM).

## Results

### Growth rates, Hb, Hb—Fe, and HME

**Fig 1:**
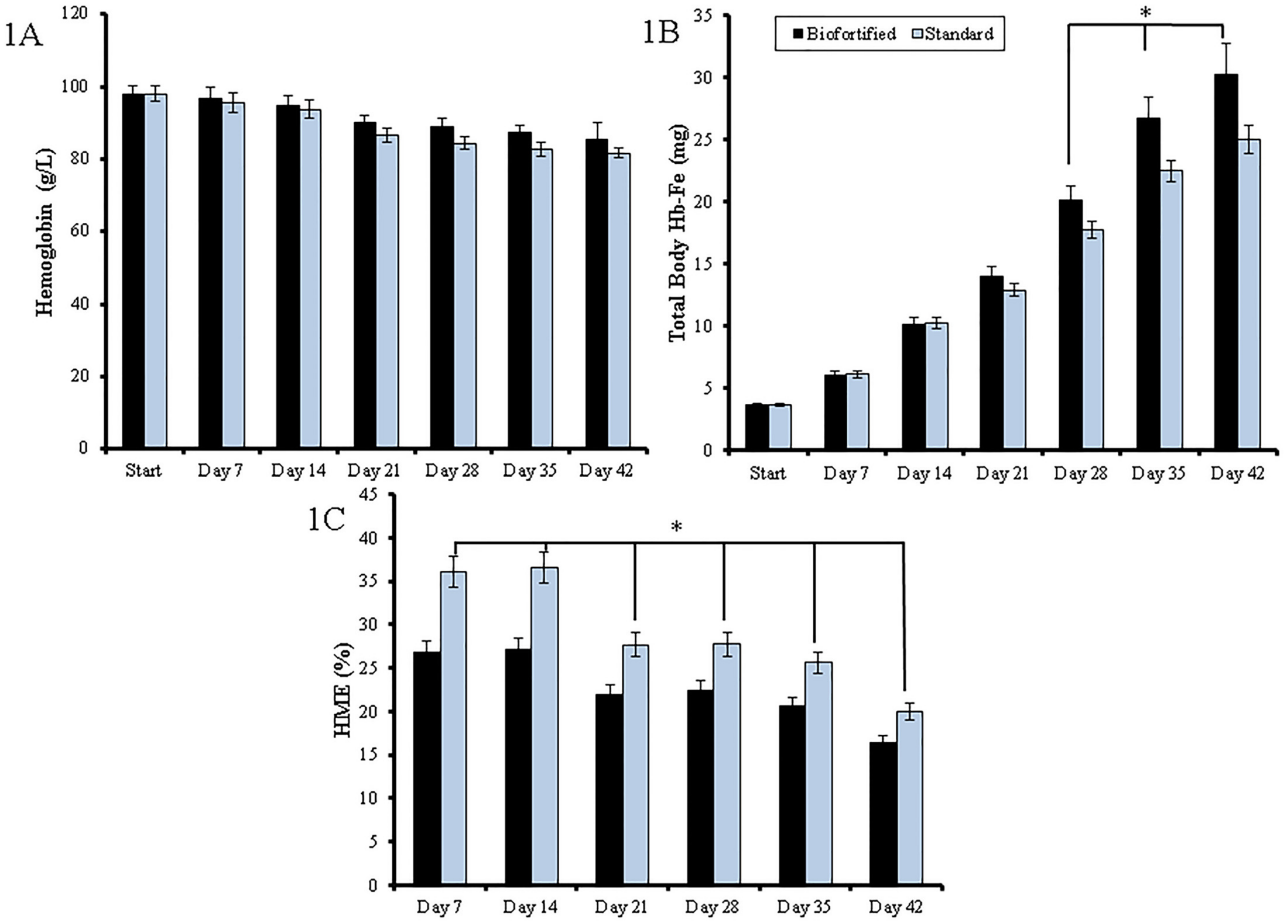
Fe-related parameters assessed during the study. (1A) Blood hemoglobin concentration (g/L), (1B) Total body Hb-Fe (mg), (1C) Hemoglobin maintenance efficiency (%). * *P* < 0.05 between treatment groups.

There were no significant differences in feed intakes at any time throughout the study (p > 0.05). However, Fe intakes were consistently higher in the group receiving the biofortified bean diet versus the group receiving the standard bean diet (p < 0.05). In addition, as from day 21 of the study, body weights were consistently higher (p>0.05) in the group receiving the Fe biofortified bean diet versus the group receiving the standard bean diet, with a significant body weight increase in the biofortified bean diet group on day 42 (p < 0.05). Although Hb concentrations were consistently elevated in the group receiving the biofortified carioca bean, this increase was not significantly different at any time point when compared to the group receiving the standard bean variety (p > 0.05, Fig 1A). Total body Hb—Fe was significantly greater in the group receiving the biofortified carioca bean during days 28–42 (p < 0.05, Fig 1B). Significant increases in HME were measured at all-time points in the group receiving the standard bean variety (p < 0.05, Fig 1C).

### Gene expression of Fe—related proteins in the duodenum

**Fig 2:**
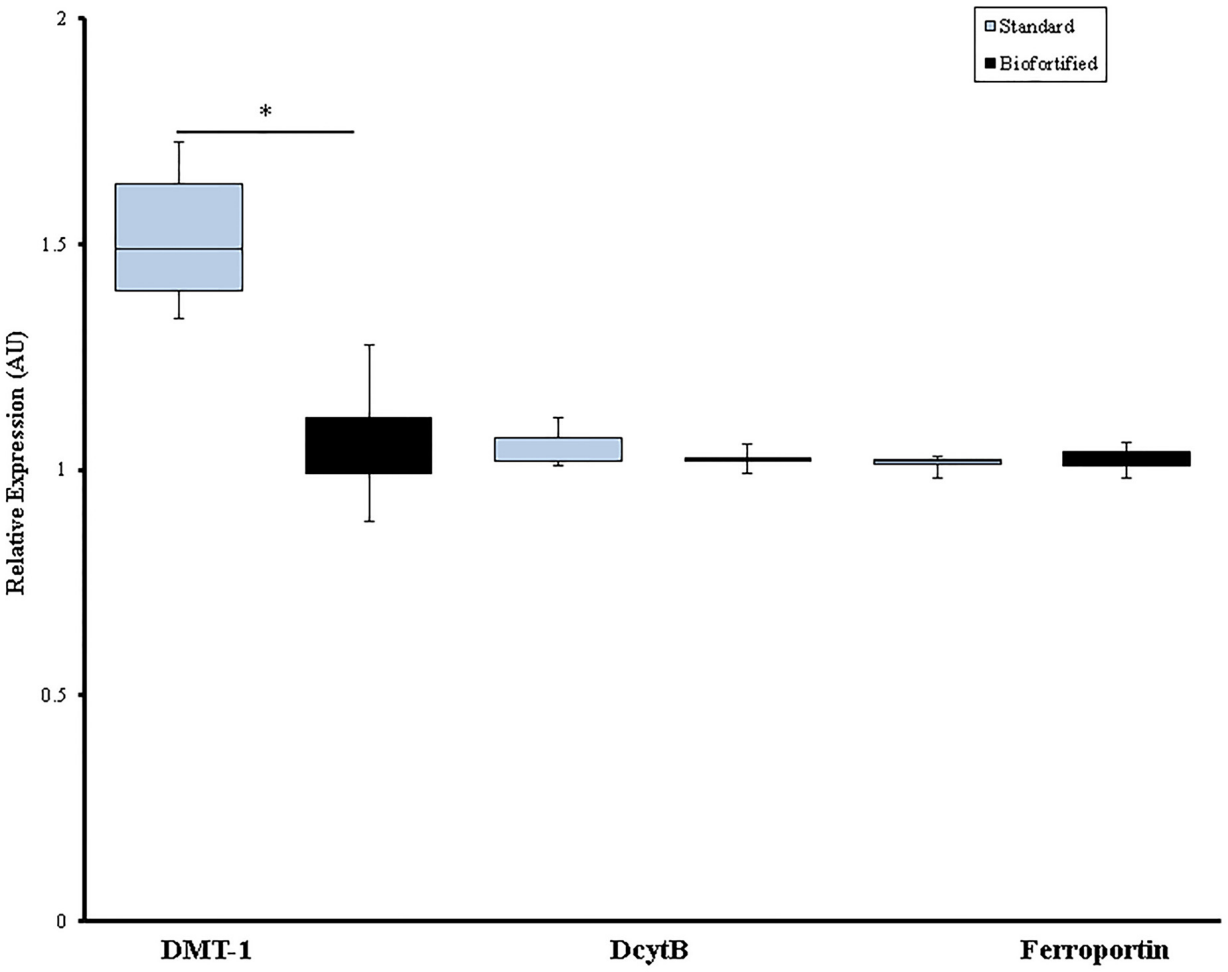
Duodenal mRNA gene expression of Fe-related proteins collected on day 42^1^. ^1^ Changes in mRNA expression are shown relative to expression of 18S rRNA in arbitrary units (AU, * *P* < 0.05).

Relative to 18S rRNA, duodenal gene expression of DMT–1 was significantly elevated in the group receiving the standard brown carioca bean diet (p < 0.05, Fig 2). However, no significant differences in DcytB and ferroportin expression were observed between treatment groups (p > 0.05, Fig 2).

### Caco–2 cell ferritin formation

Ferritin concentrations were significantly higher in cells exposed to the Fe-biofortified brown carioca bean based diet versus the standard-Fe bean based diet, as well as higher in cells exposed to the Fe-biofortified carioca bean versus the standard- Fe bean only (P < 0.05, n = 6, Table 2). These results indicate greater amounts of bioavailable Fe in the Fe-biofortified beans and diet.

**Table 2:** Ferritin concentration in *Caco-2* cells exposed to samples of beans only (whole bean), additional meal plan components and bean-based diets^1^ ^-^ ^2^.

Tested Sample^1^	Ferritin (ng/mg of protein)
Standard Fe bean only (58 μgFe/g bean)	2.86 ± 0.15^c^
Biofortified Fe bean only (106 μgFe/g bean)	4.40 ± 0.14^b^
Standard Fe Bean Based Diet (33 μgFe/g diet)	1.96 ± 0.05^e^
Biofortified Fe Bean Based Diet (48 μgFe/g diet)	2.73 ± 0.22^c^ ^d^
Basmati Rice	1.53 ± 0.10^f^
Pasta (non-enriched)	4.55 ± 0.13^b^
Potato flakes	8.26 ± 0.71^a^
Banana Chips	0.96 ± 0.12^g^
Cabbage	4.51 ± 0.16^b^
Tomato powder	3.19 ± 0.37^c^
Orange sweet potatoes	1.16 ± 0.10^g^
Cell Baseline^2^	2.53 ± 0.07^d^

^1^Caco-2 bioassay procedures and preparation of the digested samples are described in the materials and methods sections.

^2^Cells were exposed to only MEM (minimal essential media) without added

food digests and Fe (n = 6).

^a-g^ Within a column, means without a common letter are significantly different

(p < 0.05).

### Ferritin and Fe in the liver

The avian ferritin corresponds to a weight of approximately 470 to 500 kDa [2, 18, 23, 30, 31]. Although greater hepatic ferritin formation was observed in the group receiving the biofortified carioca bean diet, this increase was not significant (p > 0.05, Table 3). However, significant differences in hepatic Fe concentration was observed in the group receiving the biofortified carioca bean diet (p < 0.05, Table 3).

**Table 3:** Ferritin protein and Fe concentration in the liver.

Treatment Diet	Ferritin (μg/g wet weight)	Iron^1^ (μg/g wet weight)
Standard Fe	284±13^a^	45.5± 3.40^b^
Biofortified Fe	315±22^a^	62.6± 5.74^a^

^1^Atomic mass for iron is 55.8g/mol

^a-b^ Within a column, means without a common letter are significantly different (p < 0.05).

### Total concentration of polyphenols in the diets

The concentration of the five most prevalent polyphenolic compounds found in the bean seed coats is presented in Table 4. Both kaempferol 3–glucoside and quercetin 3–glucoside were significantly elevated in the biofortified carioca beans (p < 0.05). Kaempferol, 3,4–dihydroxybenzoin acid, and catechin were not significantly different between bean varieties (p > 0.05).

**Table 4:** Concentrations (μmol/g) of prevalent polyphenols observed in the cream seeded Carioca Beans seed coats.

Compound	Biofortified Fe	Standard Fe
3,4–dihydroxybenzoic acid	0.211±0.02^a^	0.198±0.002^a^
Catechin	0.179±0.004^a^	0.175±0.02^a^
Quercetin 3–glucoside	0.085±0.01^a^	0.00±0.00^b^
Kaempferol 3–glucoside	0.302±0.007^a^	0.206±0.008^b^
Kaempferol	0.015±0.001^a^	0.015±0.001^a^

^a,b^ Within a row, means without a common letter are significantly different (p < 0.05).

### Phytic acid concentration in the diets

Significant differences in phytate concentration were measured between biofortified and standard bean diets (n = 5, p < 0.05, Table 1). In addition, phytate:Fe ratios differed significantly between diets (15.43 ± 0.85 and 10.95 ± 0.65 for the standard bean and biofortified red mottled an bean based diets, respectively, n = 5, p < 0.05, Table 1).

## Discussion

In studies of Fe biofortification, there is a clear need and advantage to have in place screening tools capable of evaluating biofortified lines of staple food crops, both individually and in the context of the diet for which they are consumed [2, 18]. Such screening tools are also useful in identifying processing and or cooking steps that can affect Fe content and bioavailability and perhaps negate or enhance the effectiveness of the biofortified crop [33–35]. The present study, therefore evolved as an opportunity to demonstrate how the *in vitro* digestion/Caco-2 cell model and the *Gallus gallus* model of Fe bioavailability could be applied in the design of an Fe bioavailability study aimed at assessing the Fe bioavailability of Fe biofortified versus standard carioca beans. The diets that were used were specifically formulated according to the menus that were offered in the Rwandan human efficacy study [16, 17] (Table 1). Overall, the data presented in this manuscript are in agreement with previously published research [2, 18] indicating that this dual *in vitro*/ *in vivo* screening approach is effective in the assessment of Fe bioavailability of Fe biofortified beans. In the current *in vivo* assessment, and similarly with the Rwandan human efficacy trial [16, 17], the Fe biofortified bean variety presented a nutritional benefit, and delivered more absorbable Fe. Specifically and similarly to the current study (Fig 1), the results of the Rwandan human efficacy trial indicated that the Fe biofortified beans improved indicators of physical performance and Fe status, including maximum aerobic power (VO_2_max), Hb, serum ferritin, and total body Fe [11, 16, 17]. Hence, we suggest further utilizing and applying these models as screening tools to guide future human efficacy studies that assess the nutrition benefits of Fe biofortified staple crops.

Beans are a pervasive and nutritious legume grown and consumed in many parts of the world; the annual bean production in Africa is between 3–5 million metric tons [36] and is one of the top bean growing regions worldwide [37]. Specifically, the highest per capita consumption is in Kenya, Burundi, and Rwanda [37]. Beans are estimated to be the second most important source of dietary protein and the third most important source of calories in this region [14]. In terms of Fe biofortification, target levels for the bean Fe concentration have been set at nearly 95 μg/g or higher, which should likely represent a 30–50 μg/g differential from the more typical bean Fe levels [38, 39]. Hence, the Fe bioavailability assessment in a biofortified variety is particularly vital as in the present study, the differential in Fe content between the two bean lines was ~ 49 μg/g, thus confidence was high going into the study that a nutritional benefit would be observed. However, it is also important to note that Fe concentration in a harvest of beans can vary up to 15% depending on the sample size and sampling method (Tako and Glahn 2015, unpublished). This is believed to be due to the inherent variability of Fe in beans from any given variety. For example, in the sample of beans from this harvest, we measured 105 μg/g in the biofortified variety; yet from the same large harvest of beans in the weekly sampling of the beans in the associated human efficacy study, values ranged from 86 to 107 μg/g. Also, since a number of intrinsic factors, including polyphenol compounds and phytates, may influence the bioavailability of Fe from these beans and other crops [2, 10, 16, 18, 23–29, 40], increased bean Fe concentration alone may not be sufficient to yield significant physiological improvements in Fe status.

In the current study, both the *Gallus gallus* and Caco-2 cell models were used to investigate the available absorbable Fe in the biofortified carioca bean versus the standard bean variety. The *in vivo* results exemplified that even though Hb levels were slightly increased in the biofortified carioca bean group, these increases were not significant. However, significant differences in total body Hb-Fe, a sensitive biomarker of dietary Fe bioavailability [41], were observed between weeks four through six (Fig 1). Further, hepatic Fe, but not hepatic ferritin, concentration was significantly greater in the biofortified carioca bean group (Table 3). In addition, the animals receiving the standard bean variety had a higher HME at each time point when compared to the group receiving the biofortified carioca beans, indicating an adaptive response (e.g., a relative up-regulation of absorption) to less absorbable dietary Fe [2, 18, 23, 31]. As expected, mRNA gene expression analysis showed that DMT-1, a Fe homeostatic protein responsible for the transport of Fe^2+^ across the enterocyte [42], was significantly increased in the standard bean group, suggesting a compensatory mechanism in the group receiving the standard bean diet to deal with the relative reduction in the availability of absorbable Fe (Fig 2) [28, 43, 44]. The results of these physiological parameters suggest that the biofortified carioca bean diet provided more absorbable Fe to the birds, and thus yielded a slightly improved Fe status throughout the duration of the study.

Additionally, our *in vitro* assay (Table 2) further supported the *in vivo* findings. Compared to baseline, an increase in ferritin formation was observed in the cells exposed to both the biofortified beans only and the biofortified bean based diet. However, the cell ferritin values of the Fe biofortified variety were low (relative to cell baseline). These results are in agreement with previous studies aimed at assessing the Fe promoting effects of Fe biofortified black beans [18], red mottled beans [30] and pearl millet [2]. The totality of our results demonstrate that the biofortified carioca bean variety was mildly effective at increasing the absorbable Fe provided in both *in vivo* and *in vivo* experiments. Therefore, we suspected that additional compounds, such as polyphenols and/or phytic acid, present in greater quantities in the biofortified carioca beans might have hindered the expected benefit of increased Fe concentration provided by these beans.

To further investigate this, we quantified the concentration of phytic acid in both diets. It is important to note that phytic acid concentration was higher in the Fe bifortified bean variety, however, and due to the increased Fe content in the Fe biofortified bean variety, the phytic acid: Fe molar ratio was greater in the standard bean variety (p<0.05, Table 1). In addition, utilizing UPLC—MS, we sought to identify whether there were differences in the concentration of polyphenolic compounds that may limit the ability of the increased Fe in the biofortified carioca beans to provide an improved physiological benefit over the standard bean variety. Previous pre-clinical and clinical studies have strongly suggested that both phytate [16, 18, 19–22] and certain polyphenols [2, 18, 45–47], especially members belonging to the flavonoid class, have the potential to limit Fe absorption and bioavailability.

Significant differences in phytic acid concentration were observed between the biofortified and standard bean varieties (Table 1). Further, the beans seed coat polyphenols analysis detected two polyphenols that were significantly higher in concentration in the biofortified carioca bean, quercetin 3–glucoside and kaempferol 3–glucoside. In fact, measurable levels of quercetin 3–glucoside were not observed in the standard bean variety (Table 4). Previously, quercetin 3–glucoside and kaempferol 3–glucoside have been found in measureable quantities in beans [2, 18, 20, 22, 48], and were shown to complex ferric Fe (Fe^+3^), thus limiting the bioavailability of dietary Fe [49, 50]. Acute and chronic quercetin ingestion has also been shown to inhibit duodenal Fe utilization [51]. A similar response has been noted in Caco–2 cells exposed to quercetin [52]. Further, increased concentration of the flavonol kaempferol 3–glucoside has been previously detected in Fe biofortified black beans [18], and has also been shown to inhibit *in vitro* Fe bioavailability in red and pinto beans [26]. The purported mechanism for the Fe inhibitory effects of kaempferol and quercetin can be attributed to their chemical structures as they are able to chelate metallic ions, thus forming insoluble complexes with Fe^3+^ and limiting its uptake by the enterocyte [50–54]. As has been previously suggested, breeding towards an increased Fe content in beans may also increase the polyphenol, phytic acid, and similar “antinutrient” (e.g., tannins) content which in turn may limit the nutritional benefit of the Fe biofortified crops [2–4, 55]. However, since many polyphenols act as strong cellular antioxidant and anti-carcinogenic compounds [56, 57], an important goal of future research should be to identify and manipulate concentrations of specific families, even perhaps individual compounds, which display Fe inhibitory properties. Doing so, these health-promoting polyphenols may be largely retained while the effects of Fe inhibition could be limited. Overall, continued research by using the dual *in vitro* and *in vivo* screening guiding tools is needed to confirm our findings, and assess the feasibility of such a plant breeding strategy [16, 27].

## Conclusion

The current study suggests that increasing Fe concentration in the biofortified carioca beans by about 48 μg/g provides a small increase in the amount of bioavailable, and therefore absorbable, Fe. In agreement with numerous others, our data further supports the notion that phytic acid and polyphenolic compounds, such as quercetin, may likely be responsible for limiting the effects of significantly increasing Fe concentration in the biofortified beans. Since these biofortified beans increased bioavailable Fe *in vivo*, we believe that it remains a central priority to further evaluate and if possible to modify the polyphenol profile of the biofortified brown carioca bean in order for their ingestion to confer an optimal Fe status.

In addition, our findings are in agreement with the recent human efficacy trial that demonstrated that the biofortified carioca beans improved the Fe status of Rwandan women. Therefore, we suggest to further utilize the *in vitro* and *in vivo* screening tools to guide future studies aimed to assess biofortified staple food crops, as this approach will allow proceeding to human efficacy studies more effectively. In addition, these screening tools also have the capacity to cost-effectively monitor Fe biofortified crops once they are released to farmers and dispersed into the food system. Such monitoring will likely be needed to ensure the biofortification effect.

Overall, we conclude that Fe biofortified bean varieties remain a promising vehicle for increasing intakes of bioavailable Fe in African populations that consume these beans.
